# Cytotoxic Effects of Plant Sap-Derived Extracellular Vesicles on Various Tumor Cell Types

**DOI:** 10.3390/jfb11020022

**Published:** 2020-04-02

**Authors:** Kimin Kim, Hye Ju Yoo, Jik-Han Jung, Ruri Lee, Jae-Kyung Hyun, Ji-Ho Park, Dokyun Na, Ju Hun Yeon

**Affiliations:** 1 Department of Integrative Biosciences, University of Brain Education, Cheonan 31228, Korea; kimini1127@naver.com (K.K.); hyeju_yoo@naver.com (H.J.Y.); skin09_@naver.com (R.L.); 2 Department of Bio and Brain Engineering, Korea Advanced Institute of Science and Technology, Daejeon 34051, Korea; jjhan@kaist.ac.kr (J.-H.J.); jihopark@kaist.ac.kr (J.-H.P.); 3 Electron Microscopy Research Center, Korea Basic Science Institute, Cheongju 28119, Korea; jaekyung.hyun@oist.jp; 4 School of Integrative Engineering, Chung-Ang University, Seoul 06911, Korea; blisszen@lile.cau.ac.kr

**Keywords:** plant sap, extracellular vesicles, cytotoxic effects, selective toxicity, synergistic effect

## Abstract

Edible plants have been widely used in traditional therapeutics because of the biological activities of their natural ingredients, including anticancer, antioxidant, and anti-inflammatory properties. Plant sap contains such medicinal substances and their secondary metabolites provide unique chemical structures that contribute to their therapeutic efficacy. Plant extracts are known to contain a variety of extracellular vesicles (EVs) but the effects of such EVs on various cancers have not been investigated. Here, we extracted EVs from four plants—*Dendropanax morbifera*, *Pinus densiflora*, *Thuja occidentalis,* and *Chamaecyparis obtusa*—that are known to have cytotoxic effects. We evaluated the cytotoxic effects of these EVs by assessing their ability to selectively reduce the viability of various tumor cell types compared with normal cells and low metastatic cells. EVs from *D. morbifera* and *P. densiflora* sap showed strong cytotoxic effects on tumor cells, whereas those from *T. occidentalis* and *C. obtusa* had no significant effect on any tumor cell types. We also identified synergistic effect of EVs from *D. morbifera* and *P. densiflora* saps on breast and skin tumor cells and established optimized treatment concentrations. Our findings suggest these EVs from plant sap as new candidates for cancer treatment.

## 1. Introduction

Plant extracts and natural compounds from various plants and herbs have been widely used as traditional medicines, and considerable effort has been made to confirm their therapeutic effects described in ancient medicinal texts [1]. Many natural plants have been reported to possess anticancer properties, and several plant-derived compounds are currently used in cancer treatment. For example, it has been found that ginger (*Zingiber officinale*), ginkgo (*Ginkgo biloba*), and astragalus (*Astragalus membranaceus*) are helpful in attenuating the debilitating side effects of chemotherapeutic agents [2,3,4,5,6]. Plant saps, such as mastic gum, frankincense, and myrrh, are also known to possess anticancer effects, reflecting the abundance of carbohydrates and many other natural components that affect cancer [7,8,9,10,11]. Notably, most conventional anticancer agents are toxic to both malignant and normal cells, potentially leading to significant harmful side effects to healthy body tissues that can cause fatal damage [12]. Therefore, new anticancer agents with reduced side effects are in high demand [13]. 

Extracellular vesicles (EVs) play critical roles in intercellular as well as interspecies communication [14,15]. Plant-derived extracellular vesicles have been reported to have properties similar to those of mammalian exosomes and thus are capable of efficiently transferring plant components to mammalian cells to exert a therapeutic effect [16,17]. The extracellular vesicles (EVs) of plants originate from multivesicular bodies, which play a critical role in the defense response of plants to pathogen attacks at the plant cell surface [18]. For example, EVs from extracellular fluids of sunflower seedlings contain defense proteins that mediate immune responses upon infection [19]. In addition, Zhang et al. recently reported that exosome-like nanoparticles isolated from ginger (*Z. officinale*) help maintain intestinal homeostasis in mice [20]. Khomendra et al. demonstrated that nanovesicles formulated from pepper (*Bhut jolokia*) have potential as an anti-arthritic medicine [21], and Raimondo et al. isolated nanovesicles from lemon (*Citrus limon*) and showed that they exert anticancer activity [22]. Moreover, combined plant extracts and lipid nanovesicles have recently been formulated as nanovesicles, called ethosomes or phytosomes, that exhibit increased stability, facilitating their delivery for dermatological disorders [23,24]. 

In the current study, we focused on EVs contained in plant saps. These EVs are nano-sized vesicles that carry various chemical compounds and genetic materials and are capable of delivering their contents into cells with high efficiency [20]. Specifically, we investigated plant sap-derived EVs from four endemic plants collected from Bogildo and Hekseongsan in Korea: *Dendropanax Morbifera* (DM), *Pinus densiflora* (PD), *Thuja occidentalis* (TO), and *Chamaecyparis obtusa* (CO). It is known that DM and PD extracts have anticancer, antioxidant, antidiabetic, and anti-inflammatory activities, anti-melanogenic effects [25,26,27,28,29,30,31,32,33,34], whereas TO and CO extracts have antioxidant, anti-inflammatory, antimicrobial, and anti-asthmatic effects [35,36,37,38]. EVs isolated from these four kinds of plant saps were investigated for their anticancer effects on normal, low metastatic, and malignant cells. We found that DM-EVs exerted cytotoxic effects on malignant breast and skin tumor cells without affecting normal cells. PD-EVs were cytotoxic toward malignant skin tumor cells but not toward normal cells. In contrast, TO-EVs and CO-EVs showed no cytotoxic effects on most tumor cells. We also confirmed that combining DM-EVs and PD-EVs enhanced the cytotoxic effects against malignant breast and skin tumor cells over specific concentration ranges. These results indicate that EVs derived from plant sap are selectively cytotoxic against tumor cells and exert synergistic effects in combination with other EVs and potentially with conventional drugs. Thus, the use of naturally occurring EVs obtained from plants could be a new strategy to treat cancers in combination with chemical therapeutics.

## 2. Materials and Methods

### 2.1. Preparation of EVs 

Fresh sap from *D. morbifera* was collected from Bogildo, located in Wando-gun, Jeollanam-do, South Korea. *P. densiflora*, *T. occidentalis*, and *C. obtusa* saps were collected from Heukseongsan, located in Cheonan, South Chungcheong Province, South Korea. Of the 20–30 years old trees, we selected 10–20 trees that naturally secrete the sap, scratched the tree trunks with knife, and carefully collected the exuded sap with spatula. EVs were isolated from plant saps by combining 10 g of the saps with 10 mL of phosphate-buffered saline (PBS) at a 1:1 (*w*/*v*) ratio and mixing the resulting solution for 24 h in a horizontal shaker. After centrifuging the mixtures at 5000× *g* for 10 min at 4 °C, the supernatants were obtained using a 10 mL syringe, and debris was removed by filtering through a 0.22 μm membrane. Filtered samples were concentrated by centrifuging at 5000× *g* for 10 min at 4 °C using an Amicon Ultra-4 PL 100 K concentrator (Merck Millipore, Darmstadt, Germany). After centrifugation, the protein concentration of EVs was determined using a Pierce BCA (bicinchoninic acid) protein assay kit (Thermo Scientific, Rockford, IL, USA).

### 2.2. Characterizations of EVs 

The hydrodynamic size of isolated EVs was measured using dynamic light scattering (DLS) and their zeta potential was measured using a Zetasizer Nano ZS90 (Malvern Instruments, Worcestershire, UK), equipped with a solid-state laser tuned to 633 nm. Collected samples were placed at 20 °C in a thermostatic cell (Ratiolab, Hesse, Germany). The size distribution and Z-average used for determining hydrodynamic size distribution were determined by measuring scattered intensity autocorrelation functions. Thereafter, samples were mixed with distilled water at a ratio of 95:3 (*v*/*v*) for measurement of zeta potential. Diluted samples were inserted into a folded capillary cell (DTS1070; Malvern Instruments), and measurements were performed using the same equipment described above. 

For transmission electron microscopy (TEM) imaging, 5 μL of sample solution was loaded onto an EM-grid covered with a freshly glow-discharged, continuous carbon film. After allowing the sample to adsorb for 1 min, the grid was washed with droplets of deionized water and then negatively stained with 1% uranyl acetate for 1 min. Excess staining solution was blotted using a piece of filter paper, and the grid was air-dried. The sample was imaged at a focus between 0.8 μm and 1.5 μm using a JEM-1400 Plus transmission electron microscope (JEOL Ltd., Tokyo, Japan) equipped with a LaB_6_ gun and operating at 120 kV. Images were recorded using an OneView CMOS camera (Gatan, Pleasanton, CA, USA).

### 2.3. LC–MS/MS Analysis of Proteins

LC–MS/MS analysis was performed through nano ACQUITY UPLC and LTQ-Orbitrap-mass spectrometer (Thermo Electron, San Jose, CA, USA). The column used BEH C18 1.7 μm, 100 μm × 100 mm column (Waters, Milford, MA, USA). The mobile phase A for the LC separation was 0.1% formic acid in deionized water and the mobile phase B was 0.1% formic acid in acetonitrile. For tandem mass spectrometry, mass spectra were acquired using data-dependent acquisition with full mass scan (300–2000 *m*/*z*) followed by MS/MS scans. The individual spectra from MS/MS were processed using the SEQUEST software (Thermo Quest, San Jose, CA, USA) and the generated peak lists were used to query in-house database using the MASCOT program (Matrix Science Ltd., London, UK). We took only significant hits as defined by MASCOT probability analysis.

### 2.4. Cell Culture

A431 human squamous carcinoma cells (CRL-1555; American Type Culture Collection [ATCC], Rockville, MD, USA), MCF7 human breast carcinoma cells (HTB-22; ATCC, Rockville, MD, USA), and MDA-MB-231 human breast carcinoma cells (HTB-26; ATCC) were cultured in Dulbecco’s Modified Eagle Medium (DMEM; Lonza, Basel, Switzerland) supplemented with 10% fetal bovine serum (FBS; Rocky Mountain Biologicals, Missoula, MT, USA), 1% penicillin, and streptomycin (Lonza) on culture dishes. B16BL6 murine melanoma cells (high metastatic variant) (KCLB No. 8006; Korean Cell Line Bank [KCLB], Seoul, Korea) were cultured in minimum essential media alpha (α-MEM; Gibco, Invitrogen, Carlsbad, CA, USA), and B16F1 murine melanoma cells (low metastatic variant) (KCLB No. 8007; Korean Cell Line Bank [KCLB) were cultured in DMEM high glucose (Welgene, Gyeongsan, Korea) supplemented with 10% fetal bovine serum (FBS; Rocky Mountain Biologicals), 1% penicillin, and streptomycin (Lonza, Basel, Switzerland). MCF10A normal-like human breast epithelial cells (CRL-10317; ATCC, Rockville, MD, USA) were cultured in DMEM F-12 (Lonza) supplemented with 10% FBS (Rocky Mountain Biologicals), 1% penicillin, and streptomycin (Lonza). HNF human normal fibroblasts (CC-2512; Lonza) were grown in fibroblast growth medium-2 (FGM-2; Lonza). All cells were incubated at 37 °C in a humidified 5% CO_2_ environment. 

### 2.5. Cellular Uptake Measurement

DM-EVs and PD-EVs were stained with lipophilic DiI (MOP-D-3911) (Invitrogen, Waltham, MA, USA). After treating cells with different concentrations of stained EVs for 24 h, the growth medium was discarded and cells were washed three times with PBS and fixed with 4% paraformaldehyde (Wako Pure Chemical Industries, Osaka, Japan). The solution was then aspirated and Hoechst 33,342 (Invitrogen, Carlsbad, CA, USA) was added and cells were incubated at room temperature for 15 min to stain nuclei. Cells were washed with PBS containing 1% bovine serum albumen (BSA) and then imaged under a fluorescence microscope (Leica Microsystems, Wetzlar, Germany). At least three fields were selected and analyzed using Image J.

### 2.6. Cell Proliferation and Cytotoxicity Assay

The cytotoxic effects of plant sap-derived EVs were assessed by evaluating cell viability and proliferation using an EZ-Cytox cytotoxicity assay kit (Daeil Lab Service, Seoul, Korea) as described by the manufacturer. Briefly, 1 × 10^5^ cells/well in a volume of 100 uL were plated in each well of a 96-well plate and incubated at 37 °C and 5% CO_2_ for 24 h. Each well was then treated with DM-EVs, PD-EVs, TO-EVs, or CO-EVs at concentrations of 0.1, 1, 5, 10, 20, and 50 µg/mL. After a 24 h incubation, 10 µL of EZ-Cytox agent was added to each well, and the plate was incubated for 30 min to 4 h until a color change was evident. The plate was shaken gently before reading absorbance at 450 nm.

### 2.7. Endocytosis Inhibitor Assay

The pathway by which DM-EVs were internalized was analyzed using four endocytosis inhibitors: amiloride, a micropinocytosis inhibitor; chlorpromazine, a clathrin-mediated endocytosis inhibitor; cytochalasin D, a phagocytosis inhibitor; and filipin, a caveolae inhibitor. MCF10A, MDA-MB-231, and MCF7 cells were pretreated for 1 h with 10 µg/mL amiloride (Sigma-Aldrich, St. Louis, MO, USA), 10 µg/mL chlorpromazine (Sigma-Aldrich), 10 µg/mL cytochalasin D (Sigma-Aldrich), or 5 µg/mL filipin (Sigma-Aldrich) in serum-free medium. Thereafter, the medium was removed and cells were incubated with 10 µg/mL of DM-EVs in the continued presence of endocytosis inhibitors for 3 h. Cells were rinsed three times with PBS and fixed with 4% paraformaldehyde (Wako, Japan). The solution was then aspirated and cells were stained with Hoechst 33,342 (Invitrogen). Stained cells were imaged under a fluorescence microscope (Leica Microsystems) and analyzed using Image J.

### 2.8. TUNEL Assay

DNA degradation was detected by treating MDA-MB-231 cells with DM-EVs, PD-EVs, the combination of DM-EVs and PD-EVs, or cisplatin (positive control) for 24 h. Concentrations used were based on the corresponding half-maximal inhibitory concentration (IC_50_) values. Cells were fixed with 4% paraformaldehyde (Wako, Japan) and assessed using an in situ cell detection kit (Roche, Mannheim, Germany) in accordance with the manufacturer’s instructions. Cells were counterstained with Hoechst 33,342 (Invitrogen, Carlsbad, CA, USA), and images (400×) were acquired with a fluorescence microscope (Leica Microsystems).

### 2.9. Synergistic Effects Analysis

Combination indexes (CI A, x) and (CI B, x) were calculated according to Equation (1),

(1) $$\left(\mathrm{CI}\;A,x\right)=\left(\frac{\mathrm{Conc}\;A,x}{{\mathrm{IC}}_{50},A}\right),\left(\mathrm{CI}\;B,x\right)=\left(\frac{\mathrm{Conc}\;B,x}{{\mathrm{IC}}_{50},B}\right)$$

where IC_50_, A and IC_50_, B refer to the concentrations of EVs A and B, respectively, that decrease cell viability by 50%, and Conc A,x (or Conc B,x) refers to the concentration of EVs A (or B) used in combination with B (or A) that produces the same effect on viability as the individual IC_50_ value. The interaction between two types of EVs was assessed by plotting their IC_50_ values against one another using isobologram and combination index (CI) graphs. The standard line of Loewe additivity, connecting intercepts of IC_50_,A and IC_50_,B on the plot, establishes criteria for distinguishing between synergistic, additive, and antagonistic interaction such that points lying under the line indicate synergy, points on the line indicate additivity, and points above the line indicate antagonism.

### 2.10. IC_50_ Determination and Statistical Analysis

All data represent results obtained from three experiments. Statistical analyses were performed by applying two-way analysis of variance (ANOVA) followed by Dunnett’s multiple comparisons test using GraphPad Prism (GraphPad Prism Software Inc., La Jolla, CA, USA). IC_50_ values were determined by plotting concentration–response curves and extrapolating from the plot using non-linear regression analysis. *p*-values < 0.05 were considered significant; individual *p*-values are indicated in Figure legends. 

## 3. Results and Discussion

### 3.1. Characterization of Plant Sap-Derived EVs

**Figure 1:**
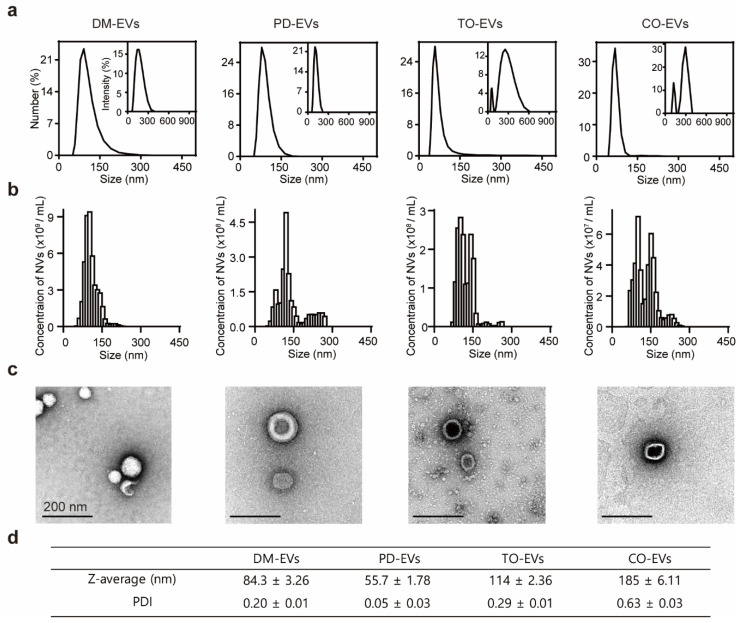
Characterization of plant sap-derived extracellular vesicles (EVs). (**a**) dynamic light scattering (DLS) measurements of particle size frequency distribution for DM-EVs, PD-EVs, TO-EVs, and CO-EVs. Insets: Intensity size distribution curves. (**b**) Nanoparticle tracking analysis (NTA) measurements of concentration and size distribution for DM-EVs, PD-EVs, TO-EVs, and CO-EVs. (**c**) TEM images of DM-EVs, PD-EVs, TO-EVs, and CO-EVs. DM, *Dendropanax morbifera*; PD, *Pinus densiflora*; TO, *Thuja occidentalis*; CO, *Chamaecyparis obtusa*. Scale bar: 200 nm. (**d**) Plant sap-derived EVs measured by Z-average and polydispersity index (PDI).

The size of EVs is considered a key parameter because it is important for cellular uptake, which together with the contents of EVs determines therapeutic efficacy. To investigate the presence and size of EVs in plant sap, we separated plant sap-derived EVs using a sequential centrifugation and filtration approach based on mammalian exosome isolation techniques. Because protein markers for plant EVs have not yet been identified, we measured protein concentration to evaluate the yield of nanoparticles. The protein concentration of DM-EVs was more than five times higher than that of PD-EVs and nearly 10-times higher than that of CO-EVs and TO-EVs (Figure S1). An analysis of the size distribution of EVs using dynamic light scattering (DLS) and nanoparticle tracking analysis (NTA) revealed that the average sizes of DM-EVs, PD-EVs, TO-EVs, and CO-EVs were 83, 56, 114, and 185 nm, respectively (Figure 1a,b). Similar to edible plant EVs, which exhibit a specific single size distribution in the range of 30–200 nm, the size of plant sap-derived EVs ranged from 80 to 200 nm, a range that was consistent across extractions [16]. TEM images revealed that the EVs were nearly spherical in shape and had a size of ~100 nm, similar to naturally occurring mammalian exosomes (50–150 nm) (Figure 1c) [39]. These EVs occasionally showed an electron-dense interior, likely reflecting infiltration of staining compounds into the vesicles [40]. The polydispersity index (PDI) values of the EVs (0.2–0.6) indicate a broad molecular weight distribution of the samples, consistent with the distribution plot (Figure 1d) [41]. 

We also analyzed protein composition of DM-EVs and PD-EVs using mass spectrometry. Selected peptides were deduced by database searching. Identified plant proteins were with respect to peroxidase for DM-EVs and cell-wall deposition protein for PD-EVs (Figure S2). Peroxidases are antioxidant enzymes responsible for releasing oxidative stresses in the cells. Oxidative stress is emerging as one of the key players in carcinogenesis, and antioxidant enzymes are known to inhibit tumor formation and prevent cancer by eliminating free radicals [42,43,44]. Cell wall deposition protein, Walls Are Thin1 protein (WAT1), is involved in secondary cell wall deposition and resistance to vascular system against pathogen invasion [45,46]. Plant cell walls are known to be correlated with anticancer activities such as the inhibition of cell proliferation, migration, and promotion of apoptosis (Figure S2) [47]. 

### 3.2. Mechanism of Cellular Uptake of EVs

To confirm the internalization of EVs into mammalian cells, we stained plant sap-derived EVs with lipophilic dye. We then treated breast cell lines with stained EVs and measured EV staining intensity per cell. We found that DM-EVs and PD-EVs were internalized to a greater extent in normal breast cells than malignant breast tumor cells (*p* < 0.0001) (Figure S3). As shown in Figure S3a, tumor cells were detached from the bottom of the plate according to the cytotoxic effect of EVs rather than normal cells after 24 h.

We hypothesized that the differences in the internalization of EVs between malignant and normal cells were related to differences in the endocytosis pathway involved [48]. Endocytosis occurs through two major mechanisms: phagocytosis and pinocytosis. Phagocytosis refers to the internalization of large particles through formation of phagosomes in cells, whereas pinocytosis corresponds to the uptake of extracellular fluid containing small molecules. Pinocytosis can be further classified into three subcategories: micropinocytosis, clathrin-mediated endocytosis, and caveolae-mediated endocytosis [49]. The uptake pathway for DM-EVs in breast cells was confirmed using four endocytosis inhibitors: cytochalasin D for phagocytosis, amiloride for micropinocytosis, chlorpromazine for clathrin-mediated endocytosis, and filipin for caveolae-mediated endocytosis. 

**Figure 2:**
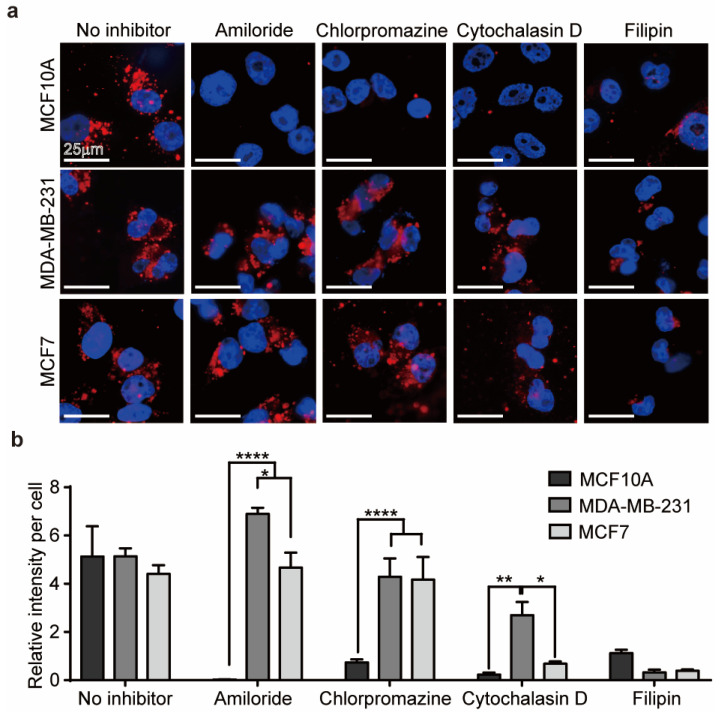
Internalization of DM-EVs in human breast cells. (**a**) Representative fluorescence microscopic images of cellular uptake of fluorescently labeled 10 µg/mL of DM-EVs for 3 h via different internalization pathways, determined using the inhibitors of 10 µg/mL amiloride (micropinocytosis), 10 µg/mL chlorpromazine (clathrin), 10 µg/mL cytochalasin D (phagocytosis), and 5 µg/mL filipin (caveolae). (**b**) Summary data showing a comparison of intracellular fluorescence intensity per cell for cells treated with different endocytic inhibitors. Data are presented as means ± standard error of the mean (SEM) (* *p* < 0.05, ** *p* < 0.01, **** *p* < 0.0001).

As shown in Figure 2a, internalization of EVs by MCF10A cells, a normal breast cell type, was significantly reduced by all of the inhibitors but was mainly affected by amiloride. Internalization of EVs by malignant MDA-MB-231 and MCF7 cells was affected by cytochalasin D and filipin but not by amiloride or chlorpromazine (Figure 2b). Therefore, DM-EVs are likely taken up into normal cells through various endocytosis pathways, whereas phagocytosis and caveolae-mediated endocytosis predominate for malignant tumor cells. The fact that malignant tumor cells internalize DM-EVs via caveolae-mediated endocytosis is also consistent with reports that overexpression of caveolin plays an important role in tumor progression and is a prognostic biomarker [50]. 

Most endocytotic pathways include a lysosomal degradative compartment, but particles internalized through caveolae-mediated endocytosis are accumulated in the cytoplasm because they are transported through the endoplasmic reticulum to the Golgi apparatus, bypassing lysosome-mediated degradation [51,52]. Because plant sap-derived EVs are internalized by malignant tumor cells through caveolae-mediated endocytosis, even if MDA-MB-231 and MCF7 cells take up less DM-EVs than MCF10A cells, they might experience greater cytotoxic effects.

### 3.3. Cytotoxic Effects of EVs against Various Cell Types

To assess the cytotoxic effects of the plant sap-derived EVs on various tumor cell types, we treated five different cell lines—MDA-MB-231 (malignant breast tumor cells), MCF7 (malignant breast tumor cells), MCF10A (normal breast cells), A431 (skin tumor cells), and HNF (normal skin cells)—with DM-EVs, PD-EVs, TO-EVs, and CO-EVs. 

We originally chose melanoma, breast, and skin tumor cells from a variety of malignant tumors because breast cancer is the most common cancer in women and skin tumor is a common invasive tumor of malignant proliferation and melanoma is reported as a source of metastasis [53,54,55]. However, unlike breast cancer and skin tumor cells, melanoma cells had no significant difference in cytotoxic effect against malignant cells compared with low metastatic cells. Therefore, we demonstrated the cytotoxic effect of DM-EVs and PD-EVs in breast and skin cells (Figure S4).

**Figure 3:**
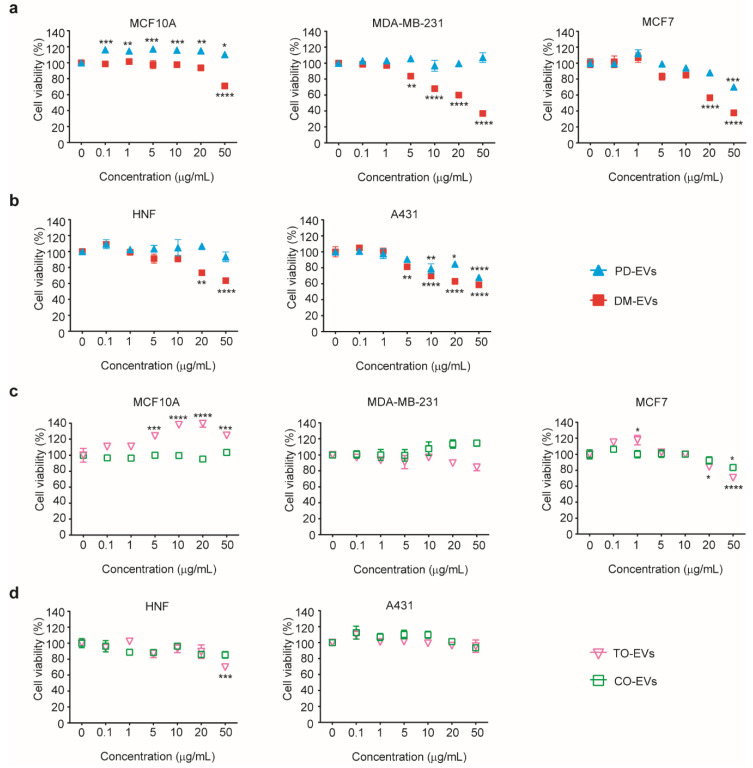
Cytotoxic effects of DM-EVs, PD-EVs, TO-EVs, and CO-EVs on five different cell types. (**a**) Viability of MCF10A, MDA-MB-231, and MCF7 human breast cells following treatment with DM-EVs or PD-EVs. (**b**) Viability of HNF and A431 human skin cells following treatment with DM-EVs or PD-EVs. (**c**) Viability of MCF10A, MDA-MB-231, and MCF7 human breast cells following treatment with TO-EVs or CO-EVs. (**d**) Viability of HNF and A431 human skin cells following treatment with TO-EVs or CO-EVs. Data are presented as means ± SEM (* *p* < 0.05, ** *p* < 0.01, *** *p* < 0.001, **** *p* < 0.0001 vs. no treat).

As shown in Figure 3a,b, cytotoxic effects of DM-EVs were detectable at 5 µg/mL for the malignant tumor cell lines, MDA-MB-231, MCF7, and A431, but not for the normal cell lines, MCF10A, and HNF. As the concentration of DM-EVs increased, cell viability gradually decreased, reaching more than 60% inhibition at 50 µg/mL in malignant tumor cells. DM-EVs showed greater cytotoxicity than PD-EVs, primarily toward malignant breast tumor cells (Figure 3a). PD-EVs exerted cytotoxic effects on both MCF7 and A431 cells, but the effect was much more prominent in A431 cells (Figure 3a,b). These results indicate that DM-EVs and PD-EVs display target-specific cytotoxicity toward malignant breast and skin tumor cells, respectively. As indicated in Figure 2, malignant tumor cells may be more sensitive to the cytotoxic effects of plant-sap derived EVs because they accumulate more of these EVs through caveolae-mediated endocytosis.

As shown in Figure 3c, TO-EVs had a very modest effect on the viability of MCF7 cells but showed no significant cytotoxicity toward other malignant tumor cells or normal cells. The viability of cells treated with TO-EVs or CO-EVs was maintained at greater than 70%, even after treatment with EV concentrations of 50 µg/mL. TO-EVs enhanced the viability of MCA10A cells compared with control. Based on these results, we selected DM-EVs and PD-EVs for subsequent synergistic studies and tumor cell-selective cytotoxicity tests.

### 3.4. Synergistic Cytotoxic Effects of DM-EVs and PD-EVs 

Combination therapy has been used to treat complex diseases, including tumors, to exploit synergistic actions of individual agents and reduce side effects [56,57]. As shown in Figure 3, DM-EVs and PD-EVs showed selective cytotoxic effects compared with TO-EVs and CO-EVs. To investigate cytotoxic effects on breast tumor and skin cancer cells, we treated with DM-EVs and PD-EVs, TO-EVs, and CO-EVs at concentration of 0.1, 1, 5, 10, 20, and 50 μg/mL in 1:1 combination, respectively, and calculated half-maximal inhibitory concentration (IC_50_) values for DM-EVs and PD-EVs alone and in combination. We chose cisplatin as a positive control because it is widely used to treat a variety of malignant tumors [58,59]. 

**Figure 4:**
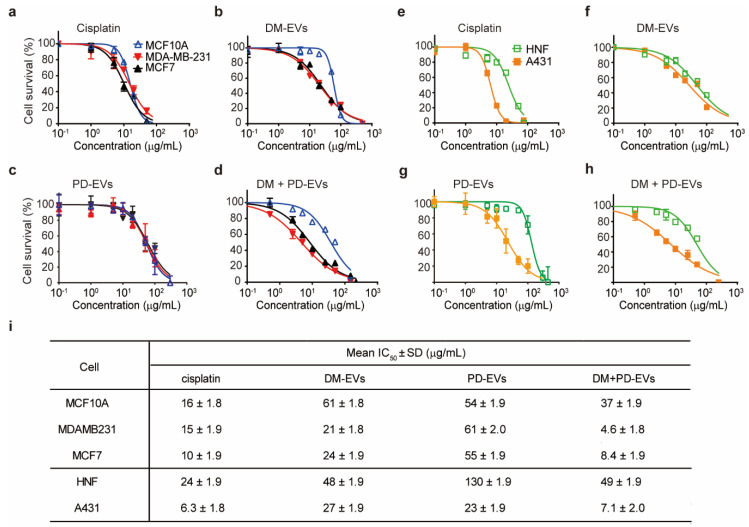
Concentration–response curves for cisplatin, DM-EVs, PD-EVs, and the combination of DM-EVs and PD-EVs against breast and skin tumor cells. (**a**–**d**) Cytotoxicity of cisplatin (**a**), DM-EVs (**b**), PD-EVs (**c**), and DM-EVs + PD-EVs (**d**) against human breast cells. (**e**–**h**) Cytotoxicity of cisplatin (**e**), DM-EVs (**f**), PD-EVs (**g**), and DM-EVs + PD-EVs (**h**) against human skin cells. (**i**) half-maximal inhibitory concentration (IC_50_) values of cisplatin, DM-EVs, PD-EVs, and the combination of DM-EVs and PD-EVs for breast and skin cells. Cisplatin was used as a positive control.

As shown in Figure 4i, the IC_50_ values of DM-EVs for MDA-MB-231 and MCF7 were lower than those for MCF10A cells, indicating that DM-EVs are more effective against malignant breast tumor cells. DM-EVs were also cytotoxic toward A431 cells but their IC_50_ value for these cells was not as low as that of cisplatin. 

PD-EVs selectively affected skin cancer cells relative to breast tumor cells, and their IC_50_ for A431 cells was lower than for HNF cells, suggesting that PD-EVs exert selective cytotoxicity toward skin cancer cells (Figure 4c,g). 

Interestingly, combined treatment with DM-EVs and PD-EVs more effectively cleared tumor cells than treatment with either EV alone (Figure 4d,h). In the case of breast cells, the IC_50_ of combined treatment with DM-EVs and PD-EVs was about 5-fold lower for MDA-MB-231 cells, 3-fold lower for MCF7 cells, and 2-fold lower for MCF10A cells compared with that for DM-EVs alone. These values were even lower than those for cisplatin. In skin cells, the IC_50_ of combined treatment with DM-EVs and PD-EVs for A431 cells was decreased by 4-fold and 3-fold, respectively, compared with DM-EVs and PD-EVs monotherapy, respectively. However, the IC_50_ value of DM-EVs and PD-EVs for HNF cells was the same for combined and individual treatment with DM-EVs and PD-EVs (Figure 4i). In addition, the IC_50_ values of DM-EVs and PD-EVs for malignant tumor cells were lower than those of cisplatin, indicating that the synergistic effects of DM-EVs and PD-EVs exceeded the cytotoxic effects of cisplatin. Notably, the IC_50_ of cisplatin was similar for both malignant and normal cells, suggesting that cisplatin could cause adverse effects in normal cells surrounding the tumor [60]. 

To study the mechanism by which combined treatment with DM-EVs and PD-EVs inhibits MDA-MB-231 cell growth, we used TUNEL (terminal deoxynucleotidyl transferase dUTP nick-end labeling) assay because the agents used to treat breast tumors are known to exert their therapeutic effects by acting through the intrinsic mitochondria apoptosis pathway [61,62,63,64]. As shown in Figure S5, the proportion of TUNEL-positive cells in EVs-treated MDA-MB-231 cells were less than 10% at respective IC_50_ values of 15 µg/mL for cisplatin, 21 µg/mL for DM-EVs, 61 µg/mL for PD-EVs, and 4.6 µg/mL for the combined DM-EVs and PD-EVs treatment. In addition, we found that only cisplatin and DM-EVs had a significant difference in cell death by apoptosis. Therefore, we assume that the main therapeutic effect of EVs is not on induction of apoptosis but growth inhibition, but further studies will be needed.

Collectively, these results indicate that combining DM-EVs and PD-EVs improves therapeutic efficacy, and because their combination was notably more effective against malignant breast and skin tumor cells, it might reduce adverse side effects.

### 3.5. Selective Cytotoxic Effect of Combined Treatment with DM-EVs and PD-EVs

**Figure 5:**
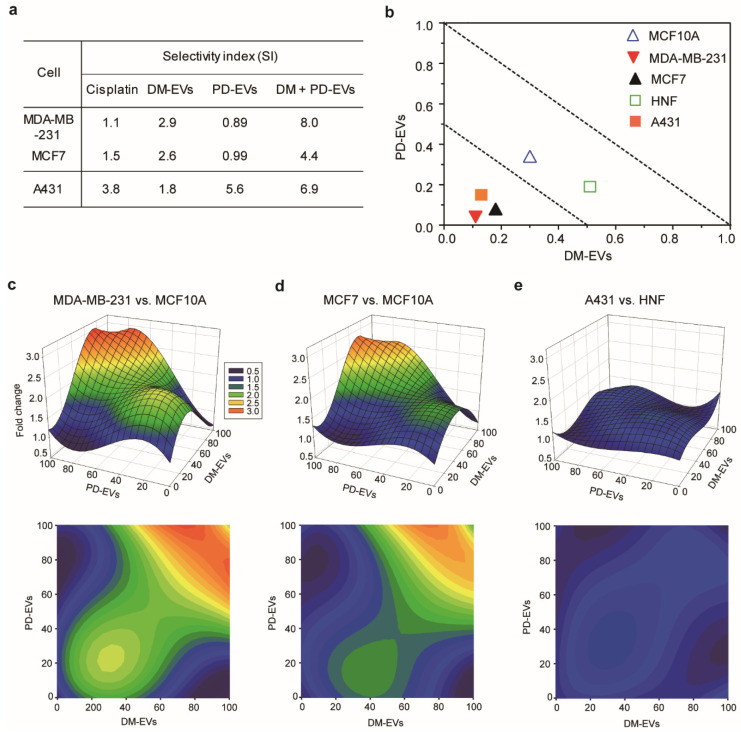
Selective index for combined treatment with DM-EVs and PD-EVs against breast and skin tumor cells. (**a**) Selective index for cisplatin, DM-EVs, PD-EVs, and the combination of DM-EVs and PD-EVs. (**b**) Isobologram curve at the 50% cell viability level for each cell type. (**c**–**e**) Selective toxicity toward malignant cells relative to normal cells, measured as fold-difference in cell survival using 3-dimensional response plots. (**c**) MDA-MB-231 versus MCF10A; (**d**) MCF7 versus MCF10A; (**e**) A431 versus HNF.

Selective cytotoxic effect is expressed as the ratio of the IC_50_ value of a treatment regimen for normal cells to the IC_50_ value for the corresponding malignant cells. This ratio, defined as the selective index (SI), is an important indicator for cytotoxic selectivity because an effective cytotoxic candidate should selectively kill malignant tumor cells with minimum adverse effects on healthy cells [65]. A high SI suggests that malignant tumor cells are more affected than normal cells, and indicates a promising cytotoxic effect. Usually, drugs with an SI > 2 are considered selective [66,67,68,69]. The SI of DM-EVs for malignant breast tumor cells compared with normal cells was about 2 and that of PD-EVs for skin cancer cells was about 5, which is higher than that of cisplatin. This indicates that DM-EVs are selective for malignant breast tumor cells and PD-EVs are selective for skin cancer cells—even more so than cisplatin (Figure 5a). 

The SI of the combination of DM-EVs and PD-EVs was higher than that of each individual treatment. The SI for combined treatment with DM-EVs and PD-EVs was 3-fold higher for MDA-MB-231 cells and 2-fold higher for MCF7 cells compared with DM-EVs alone, and higher than that for cisplatin. In the case of A431 cells, the SI of the combination was comparable to that of PD-EVs alone but was 2-fold higher than that of cisplatin. For MDA-MB-231 cells, the SI for combined treatment with DM-EVs and CO-EVs was 3.5-fold higher than that for either DM-EVs or CO-EVs alone, but it was the only combination that showed a higher SI relative to the corresponding individual treatments (Figure S6). 

We also confirmed the synergistic effect of DM-EVs and PD-EVs using isobolograms, which provide a means for assessing the interaction of a combination of two compounds based on a combination index (CI), where a CI < 0.5 indicates synergism, a CI between 0.5 to 1.0 indicates an additive effect, and a CI > 1 indicates antagonism [70,71,72,73]. In the case of MDA-MB-231, MCF7, and A431 malignant tumor cells, CIs for combined treatment with DM-EVs and PD-EVs were within the 0.5 range, whereas for MCF10A and HNF cells, CIs were between 0.5 and 1.0 (Figure 5b). These results mean that the combination of DM-EVs and PD-EVs exerted synergic effects against malignant tumor cells but not normal cells. 

Finally, we demonstrated selective cytotoxic effect by measuring changes in cell survival between malignant cells and normal cells using 3-dimensional response plots (Figure 5c–e), where *x*- and *y*-axes represent the concentrations of DM-EVs and PD-EVs, respectively, and the *z*-axis shows the fold-change in cell viability of malignant tumors cells relative to normal cells. Figure 5 shows that a 1:1 combination of DM-EVs and PD-EVs exhibited higher toxicity toward malignant breast tumor cells than normal MCF10A cells. Notably, this selective toxicity was greater for MDA-MB-231 cells than MCF7 cells (Figure 5c,d), consistent with the CI values shown in Figure 5a. This combined treatment also showed selective toxicity toward A431 cells relative to HNF cells, but the difference was modest compared with that for breast cells (Figure 5e). These results suggest that the combination of DM-EVs and PD-EVs induces selective toxicity but that the difference varies according to cell type and tissue of origin. Our future studies will evaluate the potential of various combinations of plant-derived EVs to serve as anticancer treatment regimens. 

## 4. Conclusions

Our findings suggest that DM-EVs represent a new candidate that is effective and selective toward malignant tumor cells relative to normal cells, particularly breast tumor cells. PD-EVs, on the other hand, were cytotoxic to malignant skin tumor cells but not to normal cells. Our results confirmed that tumor cells and normal cells use different endocytotic pathways to internalize DM-EVs. We hypothesized that the predominant caveolae-mediated endocytosis pathway that operates in tumor cells allows greater accumulation of DM-EVs by preventing their degradation. However, this hypothesis requires further study for a better understanding of mechanism for the endosome–lysosome pathway. We demonstrated that combined treatment with DM-EVs and PD-EVs is more effective than single treatment with DM-EVs, exhibiting synergic improvement in cytotoxic effects through the growth inhibition and apoptosis, but further study is needed to elucidate the mechanism of the therapeutic effect of EVs. We also identified concentration–response interactions between EVs and established combination cytotoxic effects through 3-dimensional analyses. Based on our results, we propose the combination of DM-EVs and PD-EVs as a potential drug regimen for treating breast and skin tumor cells with fewer side effects. Our future studies will focus on exploiting synergic effects of DM-EVs with other EVs to maximize cytotoxic effects and understanding the molecular mechanisms underlying the specificity of EV-mediated toxicity for tumor cells. 

## Supplementary Materials

The following are available online at https://www.mdpi.com/2079-4983/11/2/22/s1, Figure S1: Yield of isolated EVs per 10 g of sap. The protein concentration of DM-EVs was more that 5-times higher than that of PD-EVs, and nearly 10-times higher than that of CO-EVs and TO-EVs, Figure S2: Proteins in DM-EVs and PD-EVs identified by LC/MS. Identified plant proteins were with respect to peroxidase for DM-EVs, and cell-wall deposition protein for PD-EVs, Figure S3: Relative cellular uptake of DM-EVs and PD-EVs by human breast cells. (a) Fluorescence microscopic images of uptake of DM-EVs in human breast cells including MCF10A, MDA-MB-231, and MCF7 for 24 h. Scale bar: 100 μm, and magnified images: 75 μm. Fluorescence microscopic images of human breast cells treated with different concentrations of (b) DM-EVs and (d) PD-EVs for 24 h. Scale bar: 50 μm. Analysis of intracellular fluorescence intensity per cell internalizing (c) DM-EVs and (e) PD-EVs for each field, respectively. Mean values ± SEM (**** *P* < 0.0001). EVs were labeled with DiI (red), cells were stained with Actin Green 488 (green), and nuclear counterstaining was performed using Hoechst 33342 (blue), Figure S4: Concentration-response curves for DM-EVs against melanoma cells. The IC50 value of melanoma of malignant tumor cells had no significant difference from low metastatic cells, Figure S5: Detection of apoptotic cell death by TUNEL assay. (a) Apoptotic cells identified by TUNEL assay in MDA-MB-231 cells treated with 15 μg/mL for cisplatin, 21 μg/mL for DM-EVs, 61 μg/mL for PD-EVs, and 4.6 μg/mL for combined DM-EVs and PD-EVs at their respective IC50 values. (b) Percentage of TUNEL positive apoptotic cells in each field, respectively. Cisplatin was used as a positive control. Mean values ± SEM (* *P* < 0.05, ** *P* < 0.01 vs. no treat), Figure S6: Synergetic effect of combining DM-EVs and other EVs against skin and breast tumor cells. (a) Cytotoxicity of the combination of DM-EVs and TO-EVs against human breast cells. (b) Cytotoxicity of the combination of DM-EVs and CO-EVs against human skin cells. (c) Cytotoxicity of the combination of DM-EVs and TO-EVs against breast cells. (d) Cytotoxicity of the combination of DM-EVs and CO-EVs against human skin cells. (e) Selectivity of cytotoxicity of DM-EVs combined with TO-EVs or CO-EVs.
