# Supplementary material for: Cytotoxic Effects of Plant Sap-Derived Extracellular Vesicles on Various Tumor Cell Types
Source: J Funct Biomater. 2020 Apr 2;11(2):22. doi: 10.3390/jfb11020022 (PMC7353476; doi:10.3390/jfb11020022)
Supplement: Supplementary file 1 [file jfb-11-00022-s001.pdf]

Supplementary data for

Cytotoxic Effects of Plant Sap-Derived Extracellular Vesicles on Various Tumor Cell Types

Kimin Kim [1], Hye Ju Yoo [1], Jik-Han Jung [2], Ruri Lee [1], Jae-Kyung Hyun [3], Ji-Ho Park [2], Dokyun Na [4] and Ju Hun Yeon [1,*]

[1] Department of Integrative Biosciences, University of Brain Education, Cheonan 31228, Korea; kimini1127@naver.com (K.K.); hyeju_yoo@naver.com (H.J.Y.), skin09_@naver.com (R.L.)

[2] Department of Bio and Brain Engineering, Korea Advanced Institute of Science and Technology, Daejeon 34051, Korea; jjhan@kaist.ac.kr (J.-H.J.); jihopark@kaist.ac.kr (J.-H.P.)

[3] Electron Microscopy Research Center, Korea Basic Science Institute, Cheongju 28119, Korea; jaekyung.hyun@oist.jp

[4] School of Integrative Engineering, Chung-Ang University, Seoul 06911, Korea; blisszen@lile.cau.ac.kr

* Correspondence: jhyeon@ube.ac.kr; Tel.: +82-41-529-2621; Fax: +82-41-529-2674

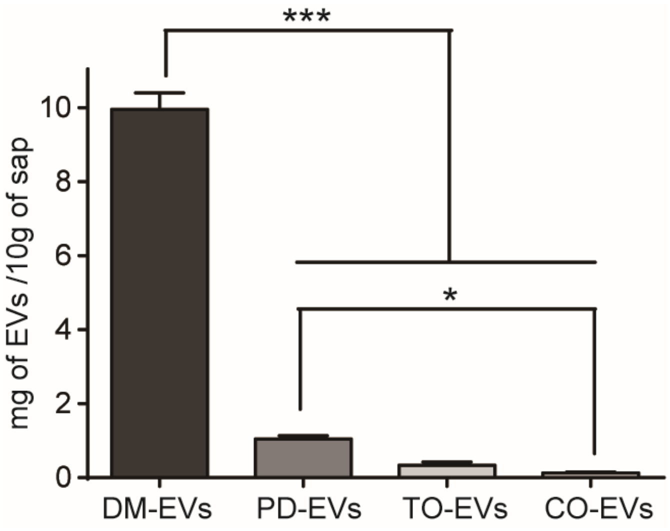

Figure S1. Yield of isolated EVs per 10 g of sap. The protein concentration of DM-EVs was more that 5-times higher than that of PD-EVs, and nearly 10-times higher than that of CO-EVs and TO-EVs.

Sample	Protein name
DM-EVs	Peroxidase
	Peroxidase 4
	Peroxidase P7-like
	Pro-hevein-like
	Peroxidase 4-like
	Peroxidase 16-like
	Polyporopepsin
	Glucan endo-1,3-beta-glucosidase
	Beta-1,3-glucanase
	Peroxidase N1-like
	Basic endochitinase A-like
PD-EVs	Protein Walls Are Thin 1-like
	Transcriptional repressor ILP1
	CLIP-associated protein-like
	Protein plastid transcriptionally active 14-like

Figure S2. Proteins in DM-EVs and PD-EVs identified by LC/MS. Identified plant proteins were with respect to peroxidase for DM-EVs, and cell-wall deposition protein for PD-EVs.

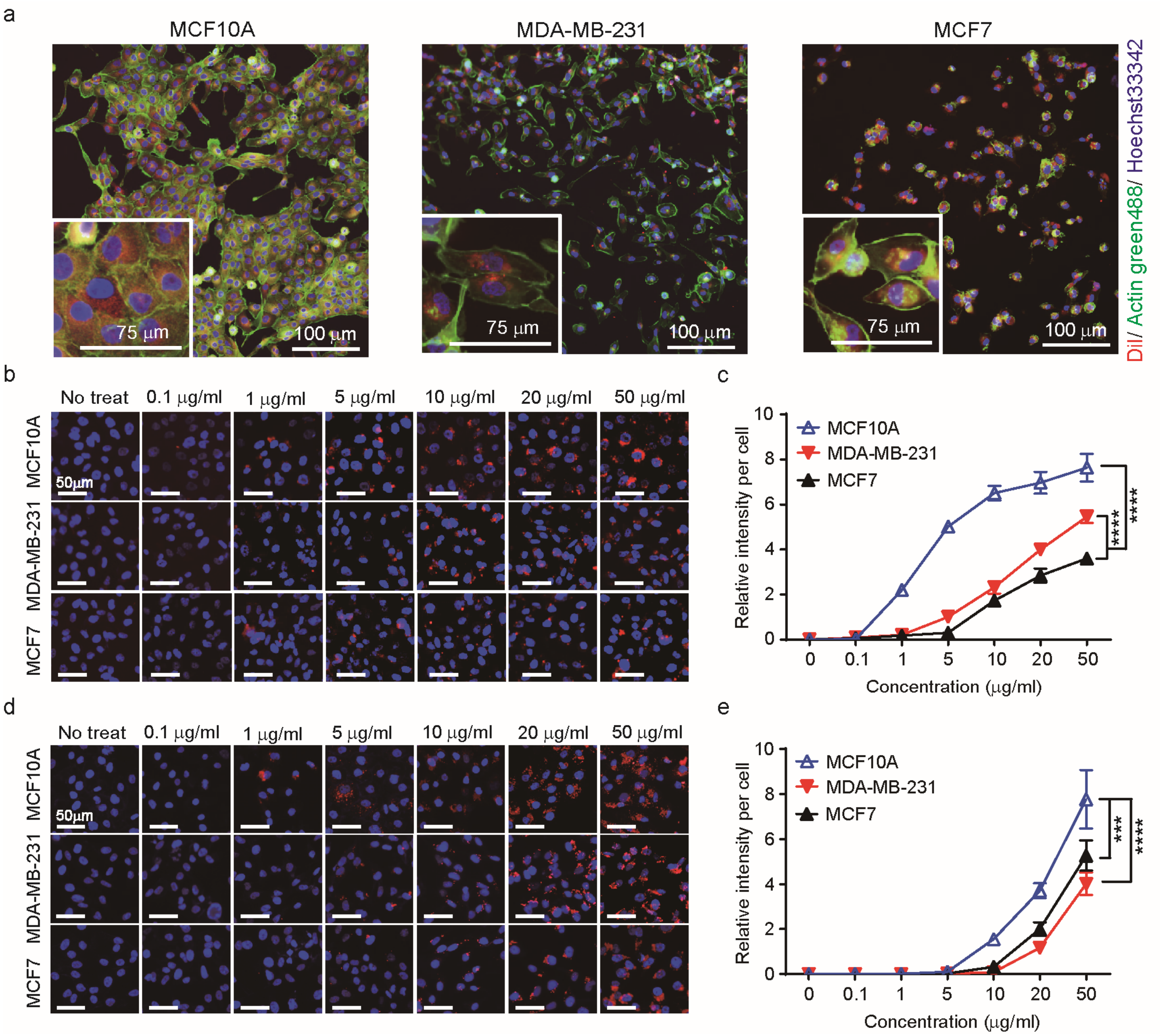

Figure S3. Relative cellular uptake of DM-EVs and PD-EVs by human breast cells. (a) Fluorescence microscopic images of uptake of DM-EVs in human breast cells including MCF10A, MDA-MB-231, and MCF7 for 24 h. Scale bar: 100 μm, and magnified images: 75 μm. Fluorescence microscopic images of human breast cells treated with different concentrations of (b) DM-EVs and (d) PD-EVs for 24 h. Scale bar: 50 μm. Analysis of intracellular fluorescence intensity per cell internalizing (c) DM-EVs and (e) PD-EVs for each field, respectively. Mean values ± SEM (*****P* < 0.0001). EVs were labeled with DiI (red), cells were stained with Actin Green 488 (green), and nuclear counterstaining was performed using Hoechst 33342 (blue).

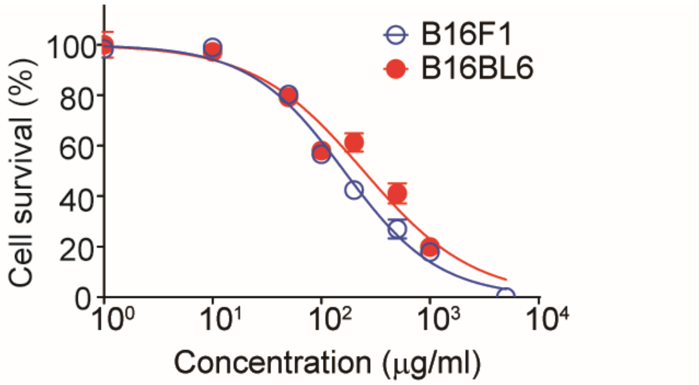

Origin	Cell	Mean IC_{50} ± SD (μg/mL)	SI
Melanoma	B16F1	16 ± 1.8	-
	B16BL6	24 ± 1.9	0.66

Figure S4. Concentration-response curves for DM-EVs against melanoma cells. The IC_{50} value of melanoma of malignant tumor cells had no significant difference from low metastatic cells.

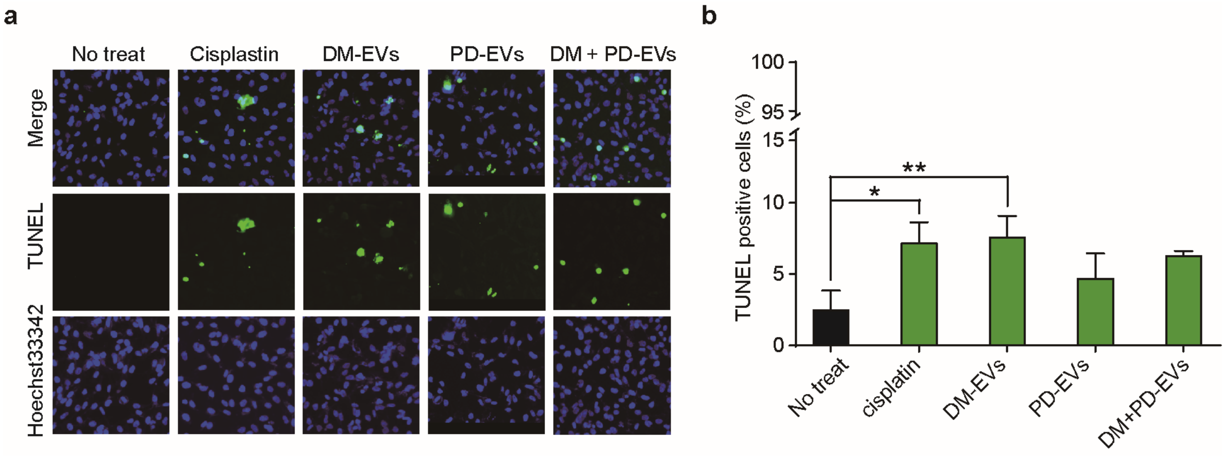

Figure S5. Detection of apoptotic cell death by TUNEL assay. (a) Apoptotic cells identified by TUNEL assay in MDA-MB-231 cells treated with 15 μg/mL for cisplatin, 21 μg/mL for DM-EVs, 61 μg/mL for PD-EVs, and 4.6 μg/mL for combined DM-EVs and PD-EVs at their respective IC_{50} values. (b) Percentage of TUNEL positive apoptotic cells in each field, respectively. Cisplatin was used as a positive control. Mean values ± SEM (**P* < 0.05, ***P* < 0.01 vs. no treat).

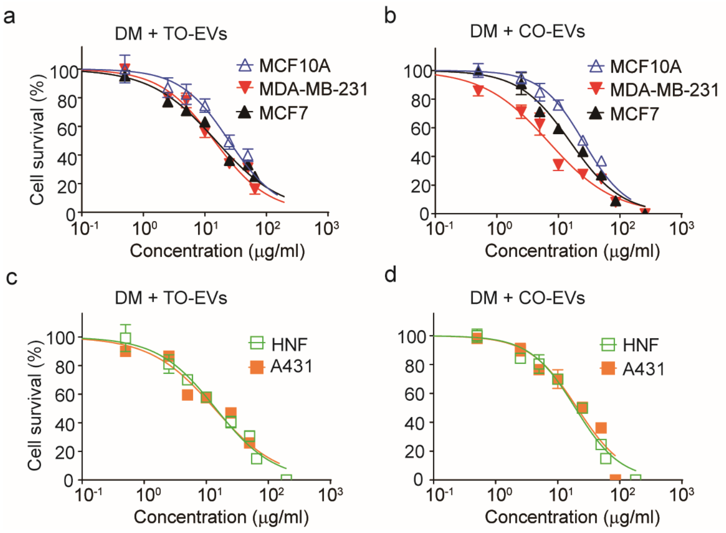

e

Cell	DM + TO-EVs	DM + CO-EVs
	Mean IC_{50} ± SD (μg/mL)	
MCF10A	25 ± 2.0	26 ± 1.9
MDA-MB -231	14 ± 1.9	7.0 ± 1.9
MCF7	15 ± 1.9	16 ± 1.9
HNF	14 ± 1.9	19 ± 1.9
A431	14 ± 1.9	21 ± 1.9
	Selectivity index (SI)	
MDA-MB -231	1.7	3.7
MCF7	1.6	1.7
A431	1.1	0.93

Figure S6. Synergetic effect of combining DM-EVs and other EVs against skin and breast tumor cells. (a) Cytotoxicity of the combination of DM-EVs and TO-EVs against human breast cells. (b) Cytotoxicity of the combination of DM-EVs and CO-EVs against human skin cells. (c) Cytotoxicity of the combination of DM-EVs and TO-EVs against breast cells. (d) Cytotoxicity of the combination of DM-EVs and CO-EVs against human skin cells. (e) Selectivity of cytotoxicity of DM-EVs combined with TO-EVs or CO-EVs.
